# Corneal Biomechanical Characteristics and Correlation Analysis in Children With Different Refractive States

**DOI:** 10.1155/joph/2450922

**Published:** 2025-01-10

**Authors:** Xiao Jing Bai, Yan Hua Wang, Tian Gang Liang, Qi Zhao, Meng Fan Cui, Jie Cheng, Wei Xiang Nie

**Affiliations:** ^1^Pediatric Ophthalmology, Shanxi Aier Eye Hospital, Aier Eye Hospital Group, Changsha, China; ^2^Pediatric Ophthalmology, Yangquan Aier Eye Hospital, Aier Eye Hospital Group, Changsha, China; ^3^Pediatric Ophthalmology, Aier Eye Hospital, Jinan University, Guangzhou, China

**Keywords:** corneal biomechanics, emmetropia, myopia, school-age children

## Abstract

**Purpose:** To investigate the correlation between corneal biomechanical characteristics and refractive status in adolescents aged 5–13 years.

**Methods:** A cross-sectional study involved 339 children aged 5–13 with a spherical equivalent (SE) range from −6.00 to +2.00 diopters. Axial length (AL) was measured by IOL Master, corneal biomechanical parameters by Corvis ST, and anterior segment parameters by Pentacam. According to SE of right eye, the subjects were divided into moderate myopia, mild myopia, and emmetropia group. The correlation between AL and SE and corneal biomechanical parameters was analyzed. The corneal biomechanical parameters of the three groups were also compared.

**Results:** The A2V value in the moderate myopia group was significantly lower than that in both the mild group and emmetropia group (*p* < 0.001). PD in the moderate group was higher than that in the mild group (*p* < 0.05), while PD in mild myopia was higher than that in emmetropia (*p* < 0.05). The SSI in the emmetropia group was significantly higher than that in the other two groups (all *p* < 0.001), and the SSI in the mild group was higher than that in the moderate group (*p* < 0.01). The A2V value in the 11–13 years old group was lower than that in the 5–7 years old group (*p* < 0.001) and 8–10 years old group (*p* < 0.01). PD in the 11–13 years old group was significantly higher than that in the 8–10 years old group (*p* < 0.001), and PD in the 8–10 years old group was significantly higher than that in the 5–7 years old group (*p* < 0.01). The SSI in the 5–7 years old group was significantly higher than that in the 8–10 years old group (*p* < 0.001), and the SSI in the 8–10 years old group was significantly higher than that in the 11–13 years old group (*p* < 0.05). AL was positively correlated with PD and negatively correlated with SSI and A2V. SE was positively correlated with A2V and SSI and negatively correlated with PD.

**Conclusions:** Corneal stiffness seems to decrease with the increase of SE. The changes of SSI, PD, and A2V were statistically significant and can be predictors of myopia progression in adolescents aged 5–13 years.

## 1. Introduction

The incidence of myopia is progressively rising. It is estimated that about one billion individuals worldwide will be at risk of vision impairment due to complications of high myopia by 2050 [1, 2]. The prevention of children with myopia from progressing to high myopia has become an urgent matter. Although the etiology of myopia remains unknown, scleral hypoxia and subsequent tissue remodeling have attracted significant attention among various theories [3–5]. The co-occurrence of axial growth and posterior scleral staphyloma in pathological high myopia suggests a strong correlation between the progression of myopia and alterations in scleral tissue. This may be attributed to the biomechanical changes in the sclera caused by the decrease in collagen content and extracellular matrix remodeling [3, 6]. Due to the testing conditions, there is no device available to measure scleral biomechanical changes in vivo currently. The assessment of corneal biomechanical changes can serve as an indirect reflection of scleral biomechanical changes, given the shared tissue origin and structural characteristics between the cornea and sclera. The utilization of this approach enables to further assess the evaluation of myopia incidence and progression [7, 8]. Previous studies have shown that alterations in corneal biomechanics can even result in changes to the blood supply to the posterior pole of the eye [9], providing additional evidence supporting the corneal–scleral association. The researchers also identified a correlation between the corneal biomechanical alterations with various degree of myopia. The studies, however, primarily focused on older children, adults, and individuals who had undergone corneal refractive surgery [10–12]. In East Asia, where the prevalence of myopia ranges from 70% to 80%, there is a paucity of studies focusing on individuals under the age of 18 [2, 13]. Therefore, we conducted an analysis of corneal biomechanical parameters obtained from Pentacam and Corvis ST measurements in children aged 5–13 years with varying degrees of myopia, aiming to identify potential corneal biomarkers associated with the development of myopia.

## 2. Materials and Methods

### 2.1. Subjects and Methods

#### 2.1.1. Study Subjects

Children visited the pediatric ophthalmology clinic at Shanxi Aier Eye Hospital from January 1 to December 31, 2021, were included in this study. Inclusion criteria: (1) aged 5–13 years; (2) best corrected visual acuity (BCVA) no less than 1.0; and (3) spherical equivalent (SE) between +2.00 and −6.00DS, astigmatism < 2.0D. Exclusion criteria: (1) intraocular pressure (IOP) over 21 mm Hg; (2) BCVA less than 1.0; (3) obvious strabismus (exotropia ≥ 15Δ, esotropia ≥ 10Δ); (4) keratoconus or other corneal diseases such as corneal trauma, corneal opacity, or postcorneal surgery; (5) organic eye diseases or history of eye surgery; (6) family history of genetic or systemic diseases; and (7) inability to cooperate with an ophthalmic examination.

Based on the spherical equivalent degree of cycloplegic refraction, subjects were divided into three groups: emmetropic group (0 ≤ SE < +2D), mild myopia group (SE ≥ −3D), and moderate myopia group (−6D ≤ SE < −3D). This study followed the Declaration of Helsinki and was approved by the hospital's ethics committee, approval number: EYETYYY-20201029-03. Informed consent was obtained from the parents or guardians of each participant.

#### 2.1.2. Research Methods

1. Comprehensive examination including standard visual assessment, slit-lamp microscopy, and fundus evaluation.
2. The optometry procedure involved the utilization of cycloplegic refraction, specifically cyclopentolate hydrochloride eye drops. Following topical anesthesia, a combination of cyclopentolate hydrochloride eye drops and compound tropicamide eye drops were administered alternately four times at five-minute intervals, followed by a resting period of 30 minutes. The initial diopter measurements were conducted using an automated Retinomax device manufactured by Nikon in Japan. The standard diopter was then obtained in accordance with the MPMVA principle, and the statistical analysis included the equivalent spherical degree of the right eye.
3. The IOL Master 700 was used to obtain five measurements of AL and corneal curvature (AL, K1, and K2), from which the average values were derived for statistical analysis.
4. The corneal biomechanical parameters were assessed using a combination of pentacam and Corvis ST corneal biomechanical analyzers. These parameters encompass the deformation amplitude ratio (DARatio2mm) and integral radius (Integral Radius), as well as the first flattening velocity (A1V) and length (A1L), second flattening velocity (A2V) and length (A2L), maximum depression radius (HCR), maximum depression peak distance (PD), first compression peacetime stiffness parameter (SP-A1), Ambrosio correlation horizontal thickness (ARTh), Corvis Biomechanics Index (CBI), corneal tomography biomechanical index (TBI), and stress–strain index (SSI).

The examinations were all conducted by professionally trained and qualified personnel at Shanxi Aier Eye Hospital.

### 2.2. Statistical Method

SPSS 26.0 was utilized for data analysis, while Graphpad Prism 8.4 was employed for graph analysis. The right eye data of each subject were extracted exclusively for analysis. Continuous measurements conforming to a normal distribution were expressed as the mean ± standard deviation, whereas those not were represented as the median (upper and lower quartiles). The corneal biomechanical parameters among the three groups were compared using ANOVA and Kruskal–Wallis tests. The correlation between AL and SE with corneal biomechanical parameters was assessed through Pearson correlation analysis and Spearman correlation analysis, respectively. *p* values less than 0.05 were regarded as statistically significant.

## 3. Results

### 3.1. Group by Equivalent Spherical

#### 3.1.1. Clinical Data

In the emmetropic group, there were 83 patients (83 eyes). The mild myopia group consisted of 152 patients (152 eyes). The moderate myopia group included 104 cases (104 eyes), as indicated in Table 1.

#### 3.1.2. Comparison of Corneal Biomechanical Parameters Among Different Myopia Groups

**Figure 1:**
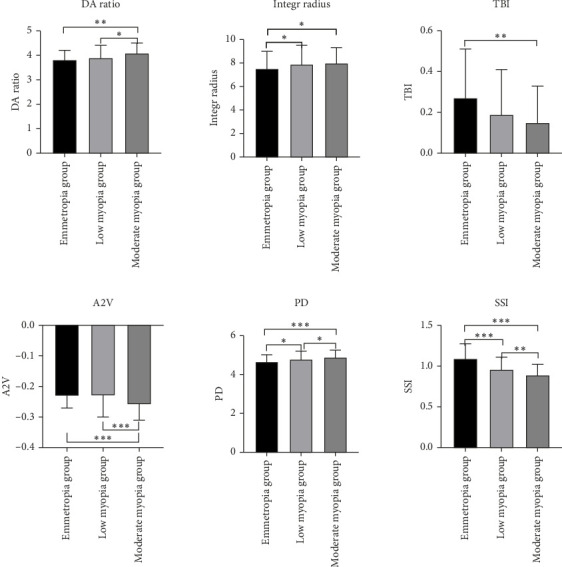
Comparison of corneal biomechanical parameters among three refractive groups.

There were no significant differences in ARTh, SP-A1, A1V, A1L, HCR, A2L, CBI, Biop, IOPnct, and CCT among the three groups (*p* > 0.05). The DA ratio was significantly higher in the moderate myopia group compared with both the mild myopia group (*p* < 0.05) and the emmetropic group (*p* < 0.01). The comprehensive radius of the emmetropic group was lower than that of both the mild and moderate myopia groups (*p* < 0.05). The TBI of the emmetropic group was highest but did not significantly differ from that of the moderate myopia group (*p* > 0.05). The A2V value in the moderate myopia group was significantly lower than that in both the mild myopia group and the emmetropic group (*p* < 0.001). PD in the moderate myopia group was higher than that in the mild myopia group (*p* < 0.01), as shown in Figure 1.

### 3.2. Group by Age

#### 3.2.1. Clinical Data

The study groups consisted of 106 patients (106 eyes) in the 5–7 years group, 157 patients (157 eyes) in the 8–10 years group, and 74 patients (74 eyes) in the 11–13 years group.  Table 2 presents the corneal biomechanical parameters of patients across different age ranges.

#### 3.2.2. Comparison of Corneal Biomechanical Parameters Among Different Age Groups

**Figure 2:**
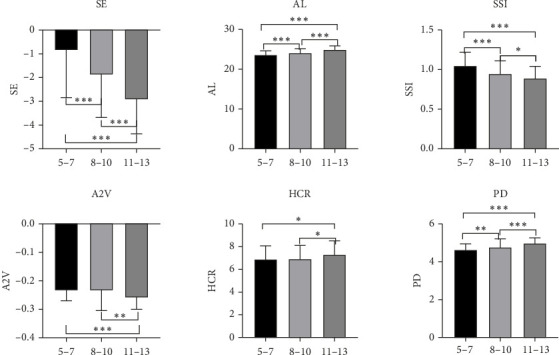
Comparison of corneal biomechanical parameters among three groups. ⁣^∗^*p* < 0.05, ⁣^∗∗^*p* < 0.01, and ⁣^∗∗∗^*p* < 0.001.

The K2, DA ratio, composite radius, ARTh, SP-A1, CBI, TBI, A1V, Biop, IOPnct, and A1L showed no significant differences among the three groups (*p* > 0.05). The results indicated a positive correlation between age and myopia degree (*p* < 0.001), as well as a positive correlation between age and axial length (*p* < 0.001). The A2V value in the 11–13 years old group was lower than that in the 5–7 years old group (*p* < 0.001) and 8–10 years old group (*p* < 0.01). Moreover, the HCR value of the 11–13 years old group was higher compared with both the 5–7 years old group and the 8–10 years old group (*p* < 0.05). In addition, PD in the 11–13 years old group exhibited a significantly higher value than that in the 8–10 years old group (*p* < 0.001), while PD in the latter was significantly higher than that in the former age range of children aged from five to seven years (*p* < 0.01). Furthermore, SSI values were found to be significantly higher in children aged from five to seven years compared with those aged from eight to ten years (*p* < 0.001), whereas SSI values were significantly higher for children aged from eight to ten compared with those aged eleven to thirteen (*p* < 0.05) (as depicted in Figure 2).

### 3.3. Correlation Between AL and Corneal Biomechanical Parameters

**Figure 3:**
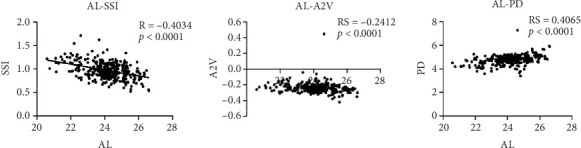
Pearson correlation between AL and corneal biomechanical parameters.

The correlation analysis revealed a positive association between AL and APTh as well as PD, with an increase in both APTh and PD corresponding to higher AL values. Conversely, there was a negative correlation observed between AL and SSI, TBI, and A2V, where an increase in AL led to a decrease in these parameters. No significant correlation was found between AL and DA ratio, composite radius, SPA1, A1V, A1L, HCR, Biop, IOPnct, CBI, or A2L (*p* > 0.05), as depicted in Figure 3.

### 3.4. Correlation Between SE and Corneal Biomechanical Parameters

**Figure 4:**
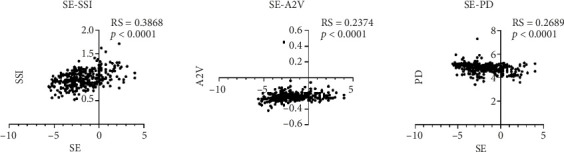
Spearman correlation between SE and corneal biomechanical parameters.

The correlation analysis results revealed positive associations between SE and TBI, A2V, and SSI. In addition, TBI, A2V, and SSI exhibited a decrease with increasing myopia degree. Conversely, SE demonstrated a negative correlation with DA ratio and PD. Consequently, both DA ratio and PD increased as myopia degree escalated. Notably, SE did not exhibit significant correlations with comprehensive radius, APTh, SPA1, CBI, A1V, A1L, Biop, IOPnct A2L, and HCR (*p* > 0.05), as depicted in Figure 4.

## 4. Discussion

Despite the absence of a dependable technique for identifying scleral biomechanical alterations in clinical environments, patients with myopia or glaucoma, which result in pathological changes in the posterior pole of the eye, also demonstrate variations in corneal biomechanical parameters. The observation of changes in corneal biomechanical parameters can serve as an indirect reflection of mechanical alterations in scleral tissue [7, 8]. In this study, we examined the corneal biomechanical parameters of school-age children aged 5–13 who were categorized based on their level of myopia. Our findings suggest that as the degree of myopia increases, there is a proportional rise in the ratio of corneal deformation amplitude to comprehensive radius, along with PD. Conversely, the A2V and SSI values exhibit a gradual decline, accompanied by statistically significant variations. The findings indicate that as myopia progresses, the corneal resistance to deformation weakens. In addition, there is a decline in both recovery capacity and corneal elasticity.

The etiology of myopia remains elusive; nevertheless, a myriad of factors can influence the elongation of the eyeball during its pathogenesis [14]. The primary causes of irreversible vision loss in pathological myopia are the elongation of the eye's axial length and the development of posterior scleral staphyloma. The researchers have investigated the correlation between pathological posterior pole dilatation of the eyeball and alterations in corneal biomechanical parameters. In the early stage of myopia, there will be changes in corneal biomechanical parameters, and as myopia progresses, these changes become more pronounced [15, 16]. The corneal biomechanical properties demonstrate a strong correlation with the refractive status [17]. This observed trend is consistent with our findings when classified according to diopter. The determination of biomechanical parameters that can precisely forecast the advancement of myopia remains inconclusive. Therefore, we have chosen SSI, A2V, and PD for further discussion based on the group observations and correlation analysis results due to their statistical significance.

SSI is a quantitative measure of corneal stiffness, which reflects the mechanical properties of corneal materials independently from their thickness. The high level of repeatability it possesses renders it a dependable indicator for evaluating corneal biomechanical properties [18–20]. The previous studies showed a significant decrease in SSI among highly myopic individuals, demonstrating an inverse correlation with the severity of myopia [21, 22]. These studies focused on adults aged over 18 years old who demonstrated a gradual development of myopia. In our study, we have observed a comparable pattern in the alteration of SSI among school-age children exhibiting rapid progression of myopia. So, we can draw a conclusion that SSI is not only correlated with the degree of myopia but also serves as an indicator for monitoring the progression of myopia to some extent. The comparison between the eyes of patients with anisometropia revealed a significantly lower SSI in the deep myopic eye compared with the fellow eye, the verification of changes in corneal biomechanics induced by myopia is also achievable [23]. Our study also observed that the SSI of the emmetropic group was significantly higher than that of the mild and moderate myopia groups. Thus, SSI could become another biological marker for this fact. It is well known that the presence of hyperopia in children is a congenital advantage when they come to combating myopia, and the presence of a thick and curved cornea may potentially function as a mechanism to mitigate the development of myopia [17]. The results of our study also demonstrated a significantly higher rate of SSI in the emmetropic group compared with the mild and moderate myopia group. In conclusion, SSI has the potential to serve as an additional biomarker for evaluating the progression of myopia following elongation of the ocular axis. It is anticipated that this discovery will garner further attention from researchers in the field.

PD refers to the measurement of the distance between the crest peaks on both sides of the nose and the temporal side during maximum compression. The higher the softness of the eyeball, the greater the PD. A2V represents the instantaneous rate of corneal vertex in the second flattening state, which serves as an indicator of the cornea's ability to recover following deformation. Smaller values correspond to poorer recovery capacity. We observed that either PD or A2V were correlated with AL and SE, and the differences between different diopter groups were found to be statistically significant. The previous studies have also identified a statistically significant correlation between these two parameters and different diopters during preoperative corneal biomechanical testing conducted on patients undergoing refractive surgery [24]. The comparison between high myopia and emmetropia also yielded consistent results [25]. The combination of our observations on school-age children leads to the conclusion that changes in PD and A2V may serve as indicators for monitoring myopia progression, enabling prediction of its development.

The corneal biomechanical parameters of school-age children aged 5–13 were also compared by the age group, revealing a gradual increase in HCR and PD with the increase of age, while A2V and SSI exhibited a gradual decrease. These differences were found to be statistically significant. In a comparative study of myopic and normal eyes aged 5–50 years, the eye tissue showed a corresponding hardening with age [26]. This is inconsistent with what we observed. The study also revealed a negative correlation between the severity of myopia and the observed decrease in ocular stiffness. The aforementioned statement aligns with the observed phenomenon of ocular softening in correlation with the progression of myopia. The correlation between corneal biomechanical properties and diopter has been consistently observed in previous studies involving preschool children [17]. The comprehensive analysis reveals that age may not be the primary cause of change but rather acts as a confounding factor in conjunction with myopia. The data also revealed a positive correlation between age and the progression of myopia. The comparison among the diopter groups also revealed that the variations in biomechanical parameters differed significantly across different diopters, and the difference was statistically significant. This is consistent with Bueno–Gimeno I's observations of white children aged 6–17 [15]. Therefore, we conducted further analysis on the correlation between AL and biomechanical parameters, as well as the correlation between SE and parameters. Our findings revealed a significant positive correlation between AL and APTh, as well as PD. In contrast, AL exhibited a significant negative correlation with SSI, TBI, and A2V. SE showed a significant positive correlation with TBI, A2V, and SSI. SE also demonstrated a significant negative correlation with the DA ratio and PD. After analyzing the correlative results, it can be concluded that as myopia progresses, the deformation amplitude of the cornea increases under external force, resulting in a slower recovery speed and decreased elasticity. Among the parameters that can reflect biomechanical properties, PD, A2V, and SSI demonstrate the most statistically significant differences.

It is widely acknowledged that there exists a correlation between IOP and corneal biomechanical parameters. However, in this study, no significant difference of Biop or IOPnct was observed neither in the groups by age nor in groups by SE. At the same time, there was no significant correlation between Biop or IOPnct and ocular axis and equivalent spherical lens. This result contrasts with some prior studies. Oscar Del Barco discovered that changes in corneal biomechanical parameters can aid early diagnosis of glaucoma among patients with high IOP [27], while Fabian Sl Yii found that children with nonpathological high myopia exhibited slower axial growth alongside increased IOP [28]. These discrepancies may be attributed to the fact that our subjects were primarily children whose intraocular pressure remained within normal limits and whose degree of myopia did not include high myopia. Thus, in the future study, we must expand the scope of subjects to enhance the generalizability of the results.

The study is subject to certain limitations. First, it is only a cross-sectional study, which can establish correlation rather than causation; second, the study subjects did not include more children with hyperopia and higher degrees of hyperopia, and it was not clear whether the changes in corneal biomechanical parameters in hyperopia children were inconsistent with those in myopia children. In future studies, we will conduct a prospective cohort study to enhance the sample size and perform further analysis on the changes observed in children with hyperopia and high myopia. Simultaneously, age will be controlled as a confounding factor to observe alterations in PD, A2V, and SSI during myopia progression. This will help elucidate whether these factors can serve as biological markers for predicting myopia progression beyond ocular axis.

## Figures and Tables

**Table 1:** Comparison of corneal biomechanical parameters among three refractive groups.

	Emmetropia	Mild myopia	Moderate myopia	*p*
AL	23.02 ± 1.07	24.30 ± 0.81	24.94 ± 0.72	< 0.001
DA ratio	3.8 ± 0.4	3.9 ± 0.5	4.1 ± 0.4	0.001
Integral radius	7.5 ± 1.5	7.9 ± 1.6	8.0 ± 1.3	0.024
ARTh	498.0 ± 146.5	535.8 ± 142.1	542.7 ± 129.3	0.066
SP-A1	114.8 ± 19.0	118.8 ± 19.6	114.6 ± 18.5	0.149
A1V	0.12 ± 0.02	0.12 ± 0.02	0.13 ± 0.02	0.276
A1L	2.46 ± 0.29	2.42 ± 0.29	2.40 ± 0.31	0.379
HCR	7.24 ± 1.20	6.91 ± 1.25	6.92 ± 1.08	0.181
SSI	1.09 ± 0.18	0.96 ± 0.15	0.89 ± 0.13	< 0.001
A2V	−0.24 (−0.26, −0.20)	−0.24 (−0.26, −0.21)	−0.26 (−0.29, −0.23)	< 0.001
A2L	1.98 (1.90, 2.14)	1.98 (1.86, 2.16)	2.00 (1.78, 2.08)	0.765
PD	4.69 (4.47, 4.93)	4.83 (4.64, 4.99)	4.95 (4.68, 5.13)	< 0.001
CBI	0.00 (0.00, 0.10)	0.00 (0.00, 0.02)	0.00 (0.00, 0.03)	0.101
TBI	0.24 (0.04, 0.40)	0.11 (0.02, 0.31)	0.08 (0.01, 0.27)	0.003
IOPnct	18.0 ± 3.7	17.8 ± 3.7	17.7 ± 3.2	> 0.05
Biop	17.6 ± 3.0	17.4 ± 3.1	17.3 ± 2.5	> 0.05

**Table 2:** Comparison of corneal biomechanical parameters among three groups.

	Age 5–7	Age 8–10	Age 11–13	*p*
SE	−0.828 ± 2.011	−1.880 ± 1.801	−2.931 ± 1.438	< 0.001
AL	23.57 ± 1.04	24.18 ± 1.01	25.04 ± 0.84	< 0.001
DA ratio	3.9 ± 0.5	3.9 ± 0.4	4.0 ± 0.4	0.858
Integral radius	7.8 ± 1.4	7.9 ± 1.6	7.7 ± 1.4	0.485
ARTh	512.7 ± 149.0	532.3 ± 129.6	551.8 ± 136.1	0.168
SP-A1	114.2 ± 18.4	118.4 ± 19.6	116.3 ± 19.2	0.205
A1V	0.12 ± 0.02	0.12 ± 0.02	0.12 ± 0.02	0.1
A1L	2.42 ± 0.28	2.43 ± 0.28	2.39 ± 0.33	0.593
HCR	6.88 ± 1.20	6.92 ± 1.17	7.30 ± 1.21	0.04
SSI	1.05 ± 0.17	0.95 ± 0.16	0.89 ± 0.15	< 0.001
A2V	−0.23 (−0.26, −0.20)	−0.24 (−0.27, −0.21)	−0.26 (−0.28, −0.24)	< 0.001
A2L	1.96 (1.88, 2.09)	1.98 (1.86, 2.17)	2.01 (1.78, 2.16)	0.517
PD	4.65 (4.47, 4.90)	4.83 (4.59, 5.02)	5.00 (4.82, 5.14)	< 0.001
CBI	0.00 (0.00, 0.10)	0.00 (0.00, 0.02)	0.00 (0.00, 0.01)	0.014
TBI	0.15 (0.02, 0.37)	0.11 (0.02, 0.29)	0.15 (0.01, 0.30)	0.493
IOPnct	17.7 ± 3.3	18.0 ± 4.0	17.6 ± 3.1	> 0.05
Biop	17.2 (15.5, 19.1)	16.5 (15.4, 18.9)	16.7 (15.8, 18.7)	> 0.05

## Data Availability

The data that support the findings of this study are available on request from the corresponding author. The data are not publicly available due to privacy or ethical restrictions.
